# Pregnancy Does Not Affect HIV Incidence Test Results Obtained Using the BED Capture Enzyme Immunoassay or an Antibody Avidity Assay

**DOI:** 10.1371/journal.pone.0013259

**Published:** 2010-10-11

**Authors:** Oliver Laeyendecker, Jessica D. Church, Amy E. Oliver, Anthony Mwatha, S. Michele Owen, Deborah Donnell, Ron Brookmeyer, Philippa Musoke, J. Brooks Jackson, Laura Guay, Clemesia Nakabiito, Thomas C. Quinn, Susan H. Eshleman

**Affiliations:** 1 Johns Hopkins University School of Medicine, Baltimore, Maryland, United States of America; 2 Statistical Center for HIV/AIDS Research and Prevention (SCHARP), Seattle, Washington, United States of America; 3 Centers for Disease Control and Prevention (CDC), Atlanta, Georgia, United States of America; 4 University of California Los Angeles, Los Angeles, California, United States of America; 5 Makerere University – Johns Hopkins University (MUJHU) Research Collaboration, Kampala, Uganda; 6 Makerere University School of Medicine, Kampala, Uganda; 7 National Institute of Allergy and Infectious Diseases, National Institutes of Health, Bethesda, Maryland, United States of America; University of Sao Paulo, Brazil

## Abstract

**Background:**

Accurate incidence estimates are needed for surveillance of the HIV epidemic. HIV surveillance occurs at maternal-child health clinics, but it is not known if pregnancy affects HIV incidence testing.

**Methods:**

We used the BED capture immunoassay (BED) and an antibody avidity assay to test longitudinal samples from 51 HIV-infected Ugandan women infected with subtype A, C, D and intersubtype recombinant HIV who were enrolled in the HIVNET 012 trial (37 baseline samples collected near the time of delivery and 135 follow-up samples collected 3, 4 or 5 years later). Nineteen of 51 women were also pregnant at the time of one or more of the follow-up visits. The BED assay was performed according to the manufacturer's instructions. The avidity assay was performed using a Genetic Systems HIV-1/HIV-2 + O EIA using 0.1M diethylamine as the chaotropic agent.

**Results:**

During the HIVNET 012 follow-up study, there was no difference in normalized optical density values (OD-n) obtained with the BED assay or in the avidity test results (%) when women were pregnant (n = 20 results) compared to those obtained when women were not pregnant (n = 115; for BED: p = 0.9, generalized estimating equations model; for avidity: p = 0.7, Wilcoxon rank sum). In addition, BED and avidity results were almost exactly the same in longitudinal samples from the 18 women who were pregnant at only one study visit during the follow-up study (p = 0.6, paired t-test).

**Conclusions:**

These results from 51 Ugandan women suggest that any changes in the antibody response to HIV infection that occur during pregnancy are not sufficient to alter results obtained with the BED and avidity assays. Confirmation with larger studies and with other HIV subtypes is needed.

## Introduction

Accurate HIV incidence estimates are critical for monitoring the HIV/AIDS epidemic, identifying populations at high risk of HIV acquisition, targeting prevention efforts, and designing and evaluating HIV prevention trials. HIV incidence can be assessed by evaluating seroconversion in longitudinal cohort studies, modeling trends in serial HIV prevalence, and applying back-calculation methods to AIDS/HIV surveillance data. However, each of those approaches has practical and methodological limitations [1], [2]. An alternative approach is to use cross-sectional surveys in combination with laboratory assays to identify recently-infected persons. However, the utility of the cross-sectional approach to HIV incidence determination has been hampered because currently available laboratory assays misclassify some chronically-infected persons as recently infected.

A variety of laboratory assays have been developed to estimate HIV incidence by cross-sectional sampling. Individuals with acute (pre-seroconversion) HIV infection can be identified by detecting HIV RNA or HIV antigen in the absence of HIV antibody [3]. However, because the window period of acute HIV infection is very short (2–3 weeks), very large populations must be tested to determine HIV incidence using that approach. An alternative approach is to determine HIV incidence using serologic assays that are designed to differentiate between individuals with recent vs. chronic HIV infection (e.g., assays that measure HIV antibody titer, avidity, isotype, specificity, or the proportion of the antibody response that is HIV-specific) [4], [5]. Those assays generally rely on use of pre-defined cut-off values to characterize HIV infections as recent vs. chronic. Unfortunately, the antibody response to HIV infection varies considerably among individuals. Chronically-infected individuals with natural or ARV-mediated viral suppression and individuals with advanced HIV disease may appear incident using some assays [6]. Misclassification of chronically-infected individuals as recently infected may also vary among different HIV subtypes [7].

In this study, we evaluated the impact of pregnancy on the performance of two serologic assays: the BED-Capture enzyme immunoassay (BED) [8] and an avidity assay based on the BioRad 1/2+ O ELISA [9]. These assays measure different characteristics of the immune response to HIV infection. The BED assay measures the proportion of antibody that is HIV-specific, while the avidity assay measures how tightly anti-HIV antibodies bind to target antigens and is not influenced by the amount or proportion of anti-HIV antibodies in a sample. These assays also differ in the type of antigens used for antibody detection and characterization. The BED assay includes antigens from subtypes B and D, as well as CRF01_AE, while the avidity assay includes antigens from a broader spectrum of HIV-1 strains, as well as antigens from HIV-2. Each of these assays is known to misclassify some chronically-infected individuals as recently infected [10], [11]. However, studies suggest that these assays may be useful for HIV incidence determination when used in combination along with non-serologic biomarkers, such as HIV viral load or CD4 cell count [6]. Effective application of these assays to cross-sectional HIV incidence determination, either alone or in multi-assay algorithms, requires knowledge of the clinical and demographic factors associated with misclassification [12]. Misclassification of chronically-infected individuals as recently infected is particularly problematic, since the proportion of individuals with chronic HIV infection in a population often greatly exceeds the proportion of individuals with recent HIV infection [12].

In low-income countries, surveillance of HIV incidence is often performed in maternal-child health clinics using serologic assays. Furthermore, cohorts of HIV seroconverters who are followed over time to determine the window periods and misclassification rates of HIV incidence assays may include women who either are or become pregnant during the observation periods. Pregnancy is associated with changes in the mother's immune system. While earlier studies suggested that pregnancy was associated with immunosuppression, more recent studies indicate that the mother's immune responses during pregnancy are actively engaged in processes related to conception, embryo implantation, and development of the placenta [13], [14]. While pregnancy does not generally influence the performance of serologic assays, the impact of pregnancy on the performance of serologic assays for HIV incidence determination has not been investigated. Changes in the dynamics of HIV infection and HIV RNA levels have been observed during pregnancy, which could also potentially influence the immunologic response to HIV infection [15]. For these reasons, we felt it was important to evaluate whether results obtained using the BED and antibody avidity assays are influenced by pregnancy.

## Materials and Methods

Plasma samples were obtained from Ugandan women who were enrolled in the HIV Network for Prevention Trials (HIVNET) 012 clinical trial. That trial compared the effectiveness of two regimens for prevention of mother-to-child transmission of HIV: a single dose nevirapine regimen and a short course zidovudine regimen [16]. A subset of women of the HIVNET 012 trial were subsequently enrolled in a 5-year follow-up study (the Extended Mother and Child Follow-up; Amendment II for HIVNET 012) [17]. Samples analyzed in this sub-study were obtained from 51 women in the HIVNET 012 follow-up study who were originally enrolled in the nevirapine arm of the HIVNET 012 trial and were stored at −80°C prior to testing. These women were infected with subtype A (N = 29), C (N = 1), D (N = 16) or intersubtype recombinant HIV (N = 4); the subtype could not be determined for one woman [18]. One-hundred thirty five plasma samples were available from the 51 women that were collected during the HIVNET 012 follow-up study: 44 samples from the 3-year visit, 48 samples from the 4-year visit, and 43 samples from the 5-year visit. For this sub-study, women were considered to have been pregnant for 9 months preceding a documented delivery in the follow-up period. The available sample set included 20 samples collected from 19 women who were pregnant at the time of sample collection during the follow-up study. Thirty-seven of the 51 women also had a baseline sample available, collected at the time of delivery or 7 days after delivery in the HIVNET 012 trial.

The BED assay was performed according to the manufacturer's instructions (Calypte Biomedical Corporation, Lake Oswego, OR, USA), using an assay cut-off of 0.8 normalized optical density units (OD-n) for recent infection (3). The avidity assay is a modified version of the Genetic Systems HIV-1/HIV-2+ O EIA (enzyme linked immunoassay, Bio-Rad Laboratories, Redmond, WA. The avidity assay was performed as previously described [6], [9], except that an assay cut-off of 40% was used for recent infection.

Generalized estimating equations were used to assess pregnancy as a predictor of OD-n levels in the BED assay. Differences in avidity assay between samples from pregnant and non-pregnant women were assessed using the non-parametric Wilcoxon rank sum test. A paired t-test was used to compare mean levels of OD-n from the BED assay among women with both pregnancy and non-pregnancy samples.

Informed written consent was obtained from all women for participation in the HIVNET 012 trial. Guidelines of the U.S. Dept. of Health and Human Services and the authors' institutions were followed in the conduct of this research. Approval for this research was obtained from the Institutional Review Boards in Uganda and at the Johns Hopkins University School of Medicine.

## Results

**Figure 1:**
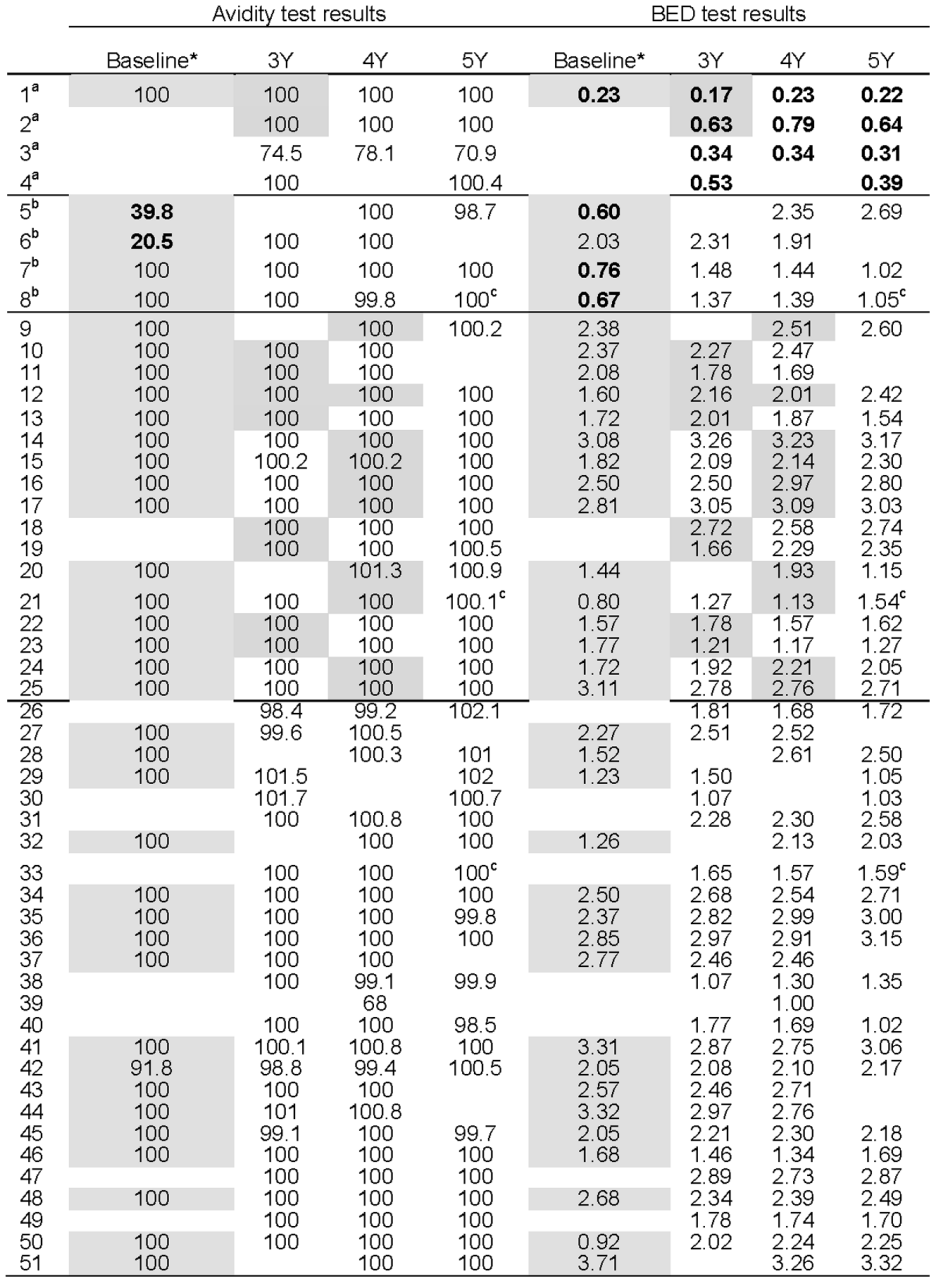
*Longitudinal samples from women in the HIV Prevention Trials Network (HIVNET) 012 trial and HIVNET 012 follow-up study were analyzed using the BED-Capture enzyme immunoassay (BED) [**8**] and an antibody avidity assay based on the BioRad 1/2 + O ELISA test (avidity) [**9**]. Baseline samples were collected near the time of delivery in the HIVNET 012 trial. Data from the baseline samples is shaded, as well as data from follow-up visits where a woman was pregnant (see Methods). Results from the BED and avidity assays that indicate recent infection (BED: results OD-n<0.8; for avidity: results <40%) are shown in bold text. Results are shown for baseline samples (obtained in the HIVNET 012 trial from the time of delivery or 7 days after delivery), and for samples collected 3, 4, and 5 years later (3Y, 4Y, 5Y). ^a^These women were identified as falsely incident at follow-up using the BED assay; two of these women had subtype A infection and two had subtype D infection. ^b^These women may have had incident infection in the HIVNET 012 trial. ^c^These samples were obtained when women were on antiretroviral therapy.

We examined whether pregnancy influenced results obtained with the BED and avidity assays. BED and avidity test results were obtained on 135 samples collected during the HIVNET 012 follow-up study (obtained 3-, 4-, and 5-years following delivery in HIVNET 012, see Methods). Twenty of the 135 results were obtained at study visits where a woman was pregnant (Figure 1); two of these results were obtained from the same woman who became pregnant twice during the HIVNET 012 follow-up study (Figure 1, #12). There was no evidence to suggest that the optical density values obtained with the BED assay when women were pregnant (n = 20 results) were different from those obtained when women were not pregnant (n = 115, p = 0.9, generalized estimating equations model). All but four of the avidity index values, including all values obtained for samples collected when women were pregnant, were in a very narrow range (between 98.4% and 101.7%); four samples collected when women were not pregnant had values between 68.0% and 78.1%. There was no evidence for a difference in avidity test results when women were vs. were not pregnant (p = 0.7, Wilcoxon rank sum). In addition, BED and avidity results were almost exactly the same in longitudinal samples from the 18 women who were pregnant at only one study visit during the follow-up study (p = 0.6, paired t-test).

We also compared results obtained at baseline (at or 7 days after delivery in the HIVNET 012 trial) to results obtained 3–5 years later (at the follow-up visits). Baseline samples tested as non-recent with the BED and avidity assays for 32 of the 37 women; five women had baseline results from the BED and/or avidity assays that were consistent with recent HIV infection (BED only: #1, #7, #8; avidity only: #6; both assays: #5). For four of those five women, test results obtained at follow-up visits were consistent with chronic HIV infection, suggesting that those four women were recently infected at the time of delivery in the HIVNET 012 trial. Of note, only one of the four women had an infant who was HIV-infected at 6 weeks of age (#8). In contrast, BED results from follow-up samples from one woman (#1) were consistent with recent HIV infection, even though the follow-up samples were collected 3–5 years after documentation of HIV infection were misclassified by the BED assay (i.e., results from follow-up samples from this woman tested as recent). False recent results were obtained by the BED assay for three other women who did not have baseline samples available for testing (#2, #3, #4). The four women who had samples that were misclassified as recent by the BED assay (#1–4, 11 samples) included two women with subtype A and two women with subtype D HIV infection. None of the follow-up samples tested in this study were misclassified as recent by the avidity assay. Based on testing of the 135 samples collected at the 3–5 year follow-up visits (i.e., in women known to have been infected for at least 3 years), 47 (92.2%) of the 51 women were correctly classified as non-recent by both assays. Three women (#8, #21, #33) initiated ARV therapy during the follow-up study; initiation of treatment did not appear to affect BED or avidity test results (Figure 1).

## Discussion

Many epidemiologic studies of HIV surveillance are conducted in maternal-child health clinics. Other studies suggest that pregnancy may modulate the immune response to infectious diseases [19]. In this study, we did not observe an effect of pregnancy on results obtained with the BED assay or an avidity assay. With the distribution of BED test results and sample size in this study, only mean differences between results obtained when women were vs. were not pregnant that exceeded an optical density of 0.37 OD-n would have achieved statistical significance (the minimum margin for non-inferiority). While we did not have the power to detect small differences in BED test results, given the range of optical density values observed in pregnancy, there was nothing to suggest that pregnancy would lead to misclassification of samples from chronically-infected women as recent. One limitation of this study is that it was performed using samples collected years earlier, and some of those samples were previously thawed and refrozen for other work. In a previous study, we found no change in BED or avidity assay results when samples were subjected up to 15 freeze-thaws, or were stored at up to 25°C for up to 15 days prior to testing [20]. Therefore, issues related to sample handling and storage of the specimens are not likely to have influenced the results obtained in this study.

The BED and avidity assay results did not change dramatically when three women initiated ARV therapy during the follow-up study. Other studies have shown that ARV therapy can affect results obtained with antibody-titer-based assays, leading to misclassification of samples from chronically infected individuals as recent [21], [22]. The three women in this study initiated ARV therapy shortly before the 5-year visit (only 5 days, 2 months, and 8 months before the visit) and therefore may not have been virologically suppressed at the time of testing. The BED assay misclassified follow-up samples from four (7.8%) of the 51 women in this study as recent, even though HIV infection was documented 3–5 years before the samples were collected; in contrast, the avidity assay did not misclassify any of the follow-up samples as recent. Misclassification of some samples as recent by the BED assay has been documented in previous studies [22], [23]. Interestingly, when a woman's samples were misclassified by BED, the misclassification was consistent over the duration of the follow-up study. Furthermore, the OD-n values (data from the BED assay) were almost identical during the period of follow-up for all 51 women (median standard deviation: 0.09, interquartile range: 0.06, 0.17). The stability of these values suggests each woman may reach an immunologic threshold and that the proportion of antibody that is HIV-specific and the avidity of anti-HIV antibodies may remain constant in many individuals for extended periods during HIV infection. Data from other studies suggests that the anti-HIV antibody response often declines when the virus is suppressed by ARV treatment or the immune system collapses in advanced HIV disease [10].

Finally, four women in this study had baseline BED and/or avidity test results that were consistent with recent HIV infection, and had follow-up test results consistent with chronic infection; those women may have been recently HIV-infected at the time of delivery in the HIVNET 012 trial. Only one of the four women transmitted HIV to her infant by 6 weeks of age. Further studies are needed to evaluate the relationship between recent HIV infection and mother-to-child transmission of HIV.

This sub-study was focused on the impact of pregnancy on the performance of the BED and avidity assays for cross-sectional HIV incidence determination. It should be noted that aside from assay misclassification, another critically important issue with cross-sectional HIV incidence determination is the whether the samples are representative of the population of interest. For example, samples drawn principally from voluntary testing and counseling centers may not be representative of the broader target population. In this regard, weighting of the samples to better reflect the target population may be helpful. The approach used by the U.S. Centers for Disease Control and Prevention (CDC) for HIV incidence determination is based on testing samples collected from HIV/AIDS surveillance systems with the BED assay [24]. The CDC attempted to improve the representativeness of those tested samples through statistical weighting [24], [25]. The CDC also determined HIV incidence with another approach, extended back-calculation, which uses surveillance reports on both AIDS cases and HIV diagnoses [24]. The CDC used these two different independent approaches to measure HIV incidence in order to corroborate findings because of the uncertainties with both approaches.

The results from this study are reassuring; they suggest that any changes in the antibody response to HIV infection that may occur during pregnancy are not sufficient to alter results obtained with the BED and avidity assays. These results indicate that pregnancy is unlikely to be a significant factor contributing to the misclassification of chronically-infected individuals as recently infected that has been observed with these assays. Some studies suggest that changes in HIV dynamics that occur during pregnancy may differ among women of different races [15]; therefore, a woman's race could theoretically affect the level or quality of anti-HIV antibodies and the performance of serologic assays for HIV incidence determination during pregnancy. The BED assay includes subtype D target antigens, but does not include subtype A antigens. In this limited study, HIV subtype was not associated with misclassification by the BED assay; two of the women who were misclassified by the BED assay had subtype A infection and two had subtype D infection. Further studies are needed to define the factors that lead to misclassification with these and other serologic assays used for HIV incidence determination and to test whether findings from our study can be generalized to other populations, including those with different prevalent HIV subtypes and strains.
